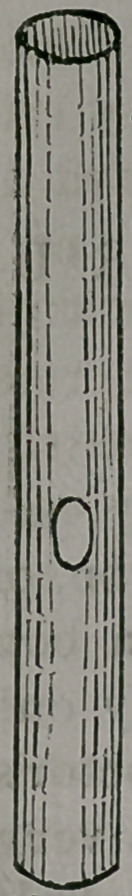# Ligation of the Femoral Artery for Traumatic Aneurism

**Published:** 1882-08

**Authors:** George J. Grimes

**Affiliations:** Columbus, Ga.


ATLANTA
### MEDICAL REGISTER.

VOL. I.] AUGUST, 1882. [No. 11

### Original.

### LIGATION OF THE FEMORAL ARTERY FOR TRAUMATIC ANEURISM.

BY GEORGE J. GRIMES, M.D., COLUMBUS, GA.

On the 12th day of September, 1880, Dr. Cameron, of this city, called to see me about Charley Mathews, a boy ten or twelve years old, who had been accidentally cut while playing with his little brother. At the Doctor's request I visited the child with him. I found him very pale and anæmic from the loss of blood, and a wound half an inch long about the middle of the inner side of the thigh. His limb was very much swollen and a pulsating tumor could be distinctly seen and felt at the point where the knife entered. In questioning the doctor about the case, I learned that the wound had been received about ten days previous, but that he did not see it at the time, nor, indeed, until seven days thereafter.

An operation was at once decided upon, and bringing the patient rapidly under the influence of chloroform, I proceeded, with the assistance of Drs. Stanford, Cameron, Johnston, and the late Dr. T. W. Grimes, to ligate the femoral artery in its fibrous canal. I preferred to tie the artery higher up, but on account of the nature of the injury, and not knowing whether the vein was wounded or not, made me a little apprehensive that there might be some complication, and hence the point selected.

An incision about four inches long was made directly over the tumor. After going through the skin and superficial fascia an immense coagulum was found dissecting its way in every direction. This was rapidly cleared away by the fingers, exposing a button-hole cut in the deep fascia and aponeurosis. The opening in the fascia was enlarged with the knife sufficiently to get out another large coagulum, when rapid hemorrhage came on. After controlling the hemorrhage the wound was well sponged out, leaving the sheath of the artery exposed with a slight wound through it, nipping out a small portion of the arterial coats. The accompanying diagram will show the shape and nature of the wound in the vessel. The vein being found uninjured an aneurismal needle armed with a ligature was passed around the artery, the needle removed and the vessel ligated. All hemorrhage ceased at once except a slight oozing from the muscular tissue.

The wound was thoroughly cleaned with carbolic acid and water, wiped dry, closed and dressed. The boy was then put to bed and his limb well wrapped with carded woolen batting. He had lost so much blood before the operation that fears were entertained that he might bleed to death on the table, but thanks to the intelligent assistance rendered by my professional friends, he went through the operation very well. He rallied slowly and was dismissed about the seventh week after the operation, the wound healing by second intention. The ligature did not come away until the sixth week.

## Figures and Tables

**Figure f1:**